# The Reflective Fostering Programme—improving the wellbeing of children in care through a group intervention for foster carers: a randomised controlled trial

**DOI:** 10.1186/s13063-021-05739-y

**Published:** 2021-11-25

**Authors:** Nick Midgley, Karen Irvine, Beth Rider, Sarah Byford, Antonella Cirasola, Poushali Ganguli, Thando Katangwe-Chigamba, Jamie Murdoch, Martin Pond, Benita Pursch, Sheila Redfern, Zena Louise Richards, Lee Shepstone, Erika Sims, Caroline Smith, Eva Sprecher, Ann Marie Swart, Solange Wyatt, David Wellsted

**Affiliations:** 1 Research Department of Clinical, Educational and Health Psychology, zUniversity College London, London, UK; 2 Centre for Health Services and Clinical Research, University of Hertfordshire, Hatfield, UK; 3 Kings College London, London, UK; 4 Anna Freud National Centre for Children and Families, London, UK; 5 Norwich Clinical Trials Unit, University of East Anglia, Norwich, UK; 6 Kent County Council, Maidstone, UK

**Keywords:** Parenting, Foster care, Children in care, Reflective parenting, Reflective Fostering Programme

## Abstract

**Background:**

The needs of children in care are a government priority, yet the evidence base for effective interventions to support the emotional wellbeing of children in care is lacking. Research suggests that supporting the carer-child relationship, by promoting the carer’s reflective parenting, may be an effective approach to improving the wellbeing of these children.

**Methods:**

The study comprises a definitive, superiority, two-armed, parallel, pragmatic, randomised controlled trial, with embedded process evaluation and economic evaluation, and an internal pilot, to evaluate the effectiveness, and cost-effectiveness, of the Reflective Fostering Programme. Randomisation is at the individual level using a 1:1 allocation ratio. The study is being conducted in local authority sites across England, and is targeted at foster carers (including kinship carers) looking after children aged 4 to 13. Consenting participants are randomly allocated to the Reflective Fostering Programme (intervention arm) in addition to usual support or usual support alone (control arm). The primary outcome is behavioural and emotional wellbeing of the child 12 months post-baseline, and secondary outcomes include the following: foster carer’s level of stress, quality of life, reflective capacity, compassion fatigue and burnout, placement stability, the quality of the child-carer relationship, child’s capacity for emotional regulation, and achievement of personalised goals set by the carer.

**Discussion:**

A feasibility study has indicated effectiveness of the Programme in improving the child-carer relationship and emotional and behavioural wellbeing of children in care. This study will test the effectiveness and cost-effectiveness of implementing the Reflective Fostering Programme as an additional aid to the support already available to local authority foster carers.

**Trial registration:**

ISRCTN 70832140.

## Administrative information

The numbers in curly brackets in this protocol refer to SPIRIT checklist item numbers. The order of the items has been modified to group similar items (see http://www.equator-network.org/reporting-guidelines/spirit-2727-statement-defining-standard-protocol-items-for-clinical-trials/).

**Table:** 

Title {1}	The Reflective Fostering Programme – improving the wellbeing of children in care through a group intervention for foster carers: a randomised controlled trial
Trial registration {2a and 2b}.	The Trial has been registered with the International Standard Randomised Controlled Trial Number registry, which includes the 24 items identified by the WHO as being the minimum dataset required for trial registration: ISRCTN 70832140
Protocol version {3}	10th November 2020. Version 3.0
Funding {4}	National Institute for Health Research - Public Health Research
-Author details {5a}	• Nick Midgley – University College London, London • Karen Irvine – University of Hertfordshire, Hatfield • Beth Rider - University of Hertfordshire, Hatfield • Sheila Redfern – Anna Freud National Centre for Children and Families, London • Sarah Byford – Kings College London, London • Poushali Ganguli - Kings College London. London • Thando Katangwe-Chigamba, University of East Anglia, Norwich • Jamie Murdoch – Kings College London, London • Martin Pond - University of East Anglia, Norwich • Benita Putsch – Anna Freud National Centre for Children and Families, London • Zena Louise Richards - University College London, London • Lee Shepstone - University of East Anglia, Norwich • Erika Sims – University of East Anglia, Norwich • Caroline Smith – Kent County Council, Maidstone • Ann Marie Swart – University of East Anglia, Norwich • David Wellsted - University of Hertfordshire, Hatfield • Solange Wyatt - University of Hertfordshire, Hatfield
Name and contact information for the trial sponsor {5b}	John Senior, University of Hertfordshire. J.m.senior@herts.ac.uk.
Role of sponsor {5c}	University of Hertfordshire is the Sponsor for this study. The Chief Investigator (CI) and Trial Manager (TM) will oversee governance and overall running of the study on behalf of the Sponsor. The Trial Steering Committee (TSC) provide overall supervision for the study on behalf of the study Sponsor and Funder. The Funder is the National Institute for Health Research.

## Introduction

### Background and rationale {6a}

The needs of children in care are a government priority [1] as they are ‘one of the most vulnerable and disadvantaged groups in our society’ [2]. Poor outcomes for children in care not only carry huge personal cost for individuals, but also increase health inequalities across society, and can place a financial burden on the state [3].

In 2019, the number of children in care in England increased by 4% to 78,150. Of these children, 72% were in foster placements [4]. Over 60% had experienced abuse or neglect prior to placement, and frequently demonstrated troubled behaviour [5]. Foster carers often struggle to respond to the complex needs of these children, leading to high levels of stress, which can affect the quality of caregiving and which may lead some to leave fostering because of burnout [6]. In turn, compromised care heightens the risk of negative outcomes for children in care, leading to increased placement instability [7] and poor health, educational, and social outcomes [8].

Formal support for foster carers in England and Wales comes primarily from the individual social worker, and this support is highly variable across different regions of the UK [9, 10]. Most parenting classes available to foster carers focus on practical behaviour management skills and do not normally explore the complex emotional needs that may underlie children’s behaviour [11]. A 2016 survey of UK foster carers concluded that local authorities fail to equip carers with the knowledge and skills needed to support children in care, especially those with emotional and behavioural difficulties [12]. A survey of over 4000 foster carers published in 2019 found that only four in 10 felt properly supported by existing support services [13]. This survey also found that the provision and take up of training for foster carers is varied across the UK, with carers highlighting a need for greater training with regard to therapeutic parenting, mental health, and attachment.

Given the importance of good quality foster care for children, there have been efforts to better support foster carers and enhance the quality of care provided [14, 15]. Yet the provision of support services in UK fostering teams is variable, and there is a lack of high-quality evidence as to which interventions are most effective [16, 17], especially when it comes to evidence of what will help foster carers best respond to the needs of primary school-aged children [9].

A review of interventions to promote the wellbeing of children in care [18] identified only four interventions for those in middle childhood that have been tested using randomised designs, none of which have a primary focus on supporting the carer-child relationship. Yet an evidence review of the fostering system for the Department for Education (DfE) [16] concluded that ‘sensitive, emotional reflective caregiving is likely to be the key experience for children and young people which will enable them to develop the qualities they need to break links between their early experiences and poor outcomes’ (p.180). ‘Reflective caregiving’ (also referred to as ‘reflective capacity’ or ‘parental reflective functioning’) refers to a caregiver’s capacity to think about their own and their child’s mental states and how these may underlie behaviour [19]. It is associated with many important facets of parenting such as sensitive caregiving, strengthened parent-child relationships, and secure attachment [20, 21]. Parents with higher reflective capacity are more able to experience difficult and emotionally activating relational exchanges without becoming overwhelmed or shutting down [22]. Research has also demonstrated that higher levels of reflective functioning can help parents tolerate distress in their children, which is also thought to be helpful in managing parenting stress [23].

Given the increasingly strong body of evidence highlighting the impact of parental reflective capacity on children’s wellbeing [24], it is unsurprising that there has been an interest in developing interventions focusing on supporting the carer-child relationship by means of promoting reflective capacity in foster carers [11, 14, 25]. Recent studies have indicated that foster carers may be especially vulnerable to losing their reflective capacity, especially when faced by stressful and demanding child-care interactions [26, 27]. Lower levels of foster carer reflective capacity have been associated with increased levels of behavioural problems in the child [28]. Helping carers manage their stress through increased reflective capacity is essential, as such stress interferes with the skills they need to help children manage their emotions [11].

Taken together, these findings support the view that the foster carer, as the most consistent relationship in the lives of children in care, should be offered help to build the skills of reflective parenting. A large body of evidence has demonstrated that achieving this leads to increased security, stability, and promotes the child’s ability to regulate and manage their own emotions [24].

Therefore, research is needed to establish whether the provision of a specialist training programme focussing on reflective parenting for foster carers, alongside usual support, would be more effective than the usual support provided by fostering teams in supporting the wellbeing of primary school-aged children in care. Further, it is imperative that local authorities have access to the high-quality evidence needed to inform budget allocation, including data regarding cost-effectiveness of support services.

#### The current project

The current project, building on two successful development and preliminary evaluation studies [29–31], aims to test the effectiveness of the Reflective Fostering Programme for foster carers, which has been designed to improve the emotional wellbeing of children in care by supporting and strengthening the carer-child relationship.

### Objectives {7}

The objectives of this study are to evaluate the effectiveness, cost-effectiveness, and process of delivery of the Reflective Fostering Programme for foster carers looking after children aged 4 to 13 years. The primary aim is to establish whether adding the Reflective Fostering Programme to usual support is more effective than usual support alone, in promoting the emotional and behavioural wellbeing of children in care; reducing levels of foster carer stress and burnout; increasing foster carer parental reflective capacity; increasing foster carer quality of life and meeting their personalised goals; improving the carer-child relationship; and in reducing placement instability.

Within the main trial, an internal pilot study will assess recruitment and randomisation procedures, examine retention and data completion rates, and explore any issues of contamination across the trial arms. In addition, an integrated economic evaluation will be conducted to determine the cost-effectiveness of implementing the Reflective Fostering Programme alongside usual support compared to usual support alone.

Across the whole trial, an embedded mixed methods process evaluation will describe how the Reflective Fostering Programme and usual support are delivered, assess intervention fidelity, understand how contextual factors shape intervention delivery, and provide explanations for the observed effects of main trial findings.

### Trial design {8}

The study comprises a definitive, superiority, two-armed, parallel, pragmatic, randomised controlled trial (RCT), with embedded process evaluation and economic evaluation, and an internal pilot, to evaluate the effectiveness, and cost-effectiveness, of the Reflective Fostering Programme. Randomisation is at the individual level using a 1:1 allocation ratio.

The trial is open-label with baseline and outcome measures collected via electronic questionnaires to ensure blinding for analysis purposes.

## Methods: participants, interventions and outcomes

### Study setting {9}

The Reflective Fostering Study is taking place in local authority fostering teams across England. The local authorities currently partnering with the study are Kent, Hertfordshire, Bristol, Devon, Lancashire, North Tyneside, Wandsworth and in north London, a consortium of the boroughs of Barnet, Camden, Islington, Hackney, Enfield, and Haringey. Additional local authorities or independent fostering agencies may be recruited, should recruitment be lower than anticipated. A full list of partnering local authorities can be obtained from the corresponding author.

### Eligibility criteria {10}

#### Study sites

Sites are selected to take part in the study based on the following criteria:

- The LA provides support to foster carers looking after children in their care
- There are suitable staff available to be trained as facilitators to run the Programme
- Social care teams are sufficiently resourced to release staff from their usual duties to facilitate the running of a group
- There are systems in place that allow the site to share information about the study with eligible foster carers and to promote the study to them
- They have access to a venue where the intervention can be delivered to foster carers (or can source a suitable venue), or have the online facilities for online delivery of the Programme
- There is someone at the site who can act as a Site Coordinator
- There are a minimum of 28 potentially eligible foster carers

#### Facilitators

The facilitators trained to deliver the intervention will come from each participating local authority. Each intervention group will be co-facilitated by two people selected by local authority fostering team managers: a social worker (or other member of the fostering support team) and a foster carer, both of whom have been trained to deliver the intervention.

#### Participants

The study population will be local authority foster carers or kinship carers (also known as ‘connected carers’) that meet the following inclusion criteria:

- The carer is currently fostering a child aged between 4 and 13 years;
- The child has been in this placement for at least 4 weeks; and
- The care plan is for the child to remain in this placement for more than 4 months.

Individuals will be excluded from the study based on the following criteria:

- Foster carer has insufficient English language ability to engage with the Programme and complete research assessments
- Foster carers where they, or their partner, have previously received the Reflective Fostering Programme

Foster carers whose partner has previously been part of the control arm (i.e. usual care) of the study may participate in a later stage of the trial, as long as their partner has completed their involvement with the study, and they are able to complete all measures in relation to a different child in their care.

To ensure health inequalities are addressed, foster carers of any child with a disability (e.g. autism, developmental delay), and those currently caring for more than one child will be eligible to take part. Foster carers who look after more than one child between the ages of 4 and 13 will be asked to choose a ‘nominated child’; that is, the one they have the greatest concerns about.

### Who will take informed consent? {26a}

Obtaining informed consent is the responsibility of the Principal Investigator at each site (referred to as the Site Lead for this study). However, the task may be delegated to a member of the research team. The Site Lead(s) will ensure that any person delegated responsibility to participate in the informed consent process is duly authorised, trained, and competent to participate according to the ethically approved protocol, principles of Good Clinical Practice (GCP) and Declaration of Helsinki.

Prospective participants will be sent a Participant Information Sheet (PIS) and invited to an information (coffee morning) meeting with the research team, at which they will have the opportunity to hear more about the study and ask questions. The plan is to hold the coffee morning at a venue central to the LA. However, if it is not possible to hold face-to-face meetings, these will become ‘virtual’ meetings conducted online through a video-conferencing platform.

Participants will have the option to provide informed consent immediately following this meeting, or to go away and consider whether they wish to go ahead and participate. Consent may be provided either on paper versions of the consent form or online via the study database. For online consent, participants will be provided with a unique username and password combination, so they can sign into the database and indicate their consent to each of the options on the form.

If the foster carers are undecided after attending the information meeting, they will be able to go away and consider participation. The Site Coordinator will contact them again to arrange for consent to be provided to a member of the research team at a later date or for their name to remain on the list for future cycles. Informed consent will always be obtained prior to the participant undergoing any study-specific procedures, including collection of baseline data. It will be made clear to foster carers that they have the right to refuse participation and are able to withdraw at any time from the trial without giving reasons, and without this affecting their usual social care support. Data collected to the point of withdrawal of consent will be retained.

### Additional consent provisions for collection and use of participant data and biological specimens {26b}

Facilitators of the Reflective Fostering Programme are considered to be study participants. This is because delivery of the Programme will be video- and/or audio-recorded and the subsequent data will be analysed in the study as part of the process evaluation. In addition, we will be holding audio-recorded focus groups with facilitators to understand experiences of programme delivery, including co-delivery by carers and social workers; and how the wider context of local authorities influenced intervention delivery. Informed consent for the Reflective Fostering sessions to be audio/video-recorded will be sought from both facilitators and foster carers, and for focus groups to be audio-recorded, from facilitators. Facilitators are expected to consent to video/audio recording as part of their role. However, facilitators will be informed that they are free to withdraw from the study at any time without being penalised in any way, but we hope that those who are trained are prepared to commit to delivery of at least one Reflective Fostering group within their local authority.

No biologic specimens will be collected.

## Interventions

### Explanation for the choice of comparators {6b}

There is no specific model of intervention or support that foster carers are routinely offered across different local authorities in England. However, all foster carers are offered some type of support from their local authority fostering support services, so this usual support was chosen as the control group.

### Intervention description {11a}

Participants will be randomised to one of two arms:

#### Intervention arm:

The Reflective Fostering Programme alongside Usual Support (where usual support is defined as the support, advice and guidance that all foster carers receive from their allocated social worker, plus any additional support services that they may receive as part of their role as a foster carer).

The Reflective Fostering Programme was developed as a group-based, psychoeducational intervention for foster carers [29]. Reflective Fostering focuses on the practical application of a set of tools that represent the principles of reflective caregiving in a shortened, highly applicable form, for foster carers of children aged 4 to 13 years. Unlike most other programmes for foster carers, it focuses on improving the carer-child relationship, by helping carers to attend to their own state of mind and experiences, and providing carers with practical ways to help build and maintain supportive relationships with the children in their care [30].

The Programme uses both psychoeducation and practical activities that link directly to the foster carers’ own experiences. The aim is to enhance the capacity of foster carers to be mindful of the impact that caring for the child has on their own thoughts and feelings, and the influences of their current state of mind on their reaction to their foster child, which in turn helps them to become more open and curious about the thoughts, feelings, and experiences of the child. When carers are helped to do this, they can better manage their own feelings and respond more effectively to the needs of the child in their care.

The Reflective Fostering Programme involves ten 2–3-h sessions offered to a group of 6–10 foster carers over a 12–14-week period.

There is a specific focus for each session:

1. Introduction to the Reflective Fostering Programme
2. Reflecting on yourself as a foster carer: The Carer Map
3. Seeing and Thinking about your foster child in different ways
4. Understanding and helping your foster child who has experienced developmental or other trauma
5. Trust, relationships, and helping your foster child get on better with other people
6. Responding to problematic behaviour in a reflective way
7. Understanding misunderstandings: putting your Carer map and APP together
8. Getting the help and support you need as a foster carer - family, friends, and the team around you
9. Moving on—getting ready for the end of the Reflective Fostering Programme
10. Review and ending session: how to keep the model in mind and stay feeling supported

Groups are delivered by trained facilitator pairings (one registered Social Care worker and one experienced foster carer), who are provided with a weekly consultation from specialists at the Anna Freud National Centre for Children and Families (AFNCCF). The model of co-delivery of the Reflective Fostering Programme by experienced foster carers alongside LA social workers builds on the success of this approach in ‘Skills to Foster’ training [32]. The co-delivery approach can build competence and capability within the system and expands the holding of ‘expertise’ within the system’s network.

After the Programme ends, carers are provided with access to materials about Reflective Fostering online and are encouraged to form an online support group.

#### Control arm: usual support alone

Currently there is considerable variability in the kind of additional support services that may be available. Some local authorities offer structured training and support networks, with possibility of referral to specialist services, but to our knowledge none currently offer a programme like the Reflective Fostering Programme, based on the principles of ‘reflective parenting’. The process evaluation will document what ‘usual support’ includes, and variation in usual support will be mapped onto variation in the primary outcome of the study.

### Criteria for discontinuing or modifying allocated interventions {11b}

All participants will be explicitly made aware of their rights to withdraw from the study and how to do this. This will be made clear to the participant within the information sheet and at the point of consent. Participants may withdraw from the study (before, during or after delivery of the Reflective Fostering Programme, and not agree to data collection) or withdraw from the intervention (during the Reflective Fostering Programme but agree to continue data collection). Although not obliged to give a reason for discontinuing in the study, a reasonable effort will be made to establish this reason, whilst remaining fully respectful of the participant’s rights.

Once the intervention has started, those in the intervention arm who choose to withdraw from the study but wish to continue attending the Reflective Fostering Programme may do so, as long as they still give consent for sessions to be video-recorded.

### Strategies to improve adherence to interventions {11c}

All facilitators of the intervention will be offered regular consultation meetings with a member of the team that developed the Reflective Fostering Programme, to help support adherence to the Programme delivery. Participants who do not attend a session of the Reflective Fostering Programme without notice will be contacted by one of the facilitators, to identify any obstacles and to encourage attendance. Unless the participant withdraws from the study, facilitators will make contact after each missed session, usually by email or text message, depending on usual practice in each local site.

### Relevant concomitant care permitted or prohibited during the trial {11d}

All foster carers will continue to receive usual support from their local authority whilst they are in the study, including any specialist training or support. If carers or the child in their care are currently receiving any other form of counselling or mental health support, this will continue, and they may also be referred for specialist support (e.g. to CAMHS or Educational Psychology) as usual, without affecting their participation in the study. Attendance at the Reflective Fostering Programme is not designed to replace such usual support.

### Provisions for post-trial care {30}

Participants randomised to the control arm may have the opportunity to attend the Reflective Fostering Programme after the study is finished, if the local authority choose to continue offering the Programme. Neither the NIHR nor the study Sponsor will be responsible for funding any intervention post-trial.

### Outcomes {12}

#### Primary outcome

The primary outcome for this study is the absolute value of the behavioural and emotional wellbeing of the child 12 months post-baseline, as provided by their score on the carer-report Strengths and Difficulties Questionnaire (SDQ) [33].

#### Secondary outcomes

Secondary outcomes are the absolute values for the SDQ at 4 months, and the following outcome measures recorded at 4 and 12 months post-baseline: foster carer’s level of stress as measured by the Parenting Stress Index (short form) (PSI 4-SF) [34]; foster carer’s quality of life and foster carer’s compassion fatigue and burnout (Professional Quality of Life Questionnaire, PRoQOL) [35]; the Child’s capacity for emotional regulation, as measured by the Emotional Regulation Checklist (ERC) [36]; foster carer’s reflective capacity, as measured by the Parental Reflective Functioning Questionnaire (PRFQ) [37]; achievement of personalised goals set by the foster carer (measured by the Carer Defined Problem Scale (CDPS)) [38]. In addition, we will examine placement stability, which is recorded in relation to changes of social worker, change of school, or placement change, and reasons for any change. The data will be recorded using the Placement Stability Log.

See section ‘Plans for assessment and collection of outcomes {18a}’ for a more detailed description of the study outcome measures.

#### Economic evaluation measures:

Economic measures include a version of the Child and Adolescent Service Use Schedule (CA-SUS) designed and tested for use with Children in Care in an earlier feasibility study [31, 39] and the Child Health Utility 9 Dimensions (CHU9D) measure of health-related quality of life [40], proxy reported by foster carers.

### Participant timeline {13}

**Fig. 1:**
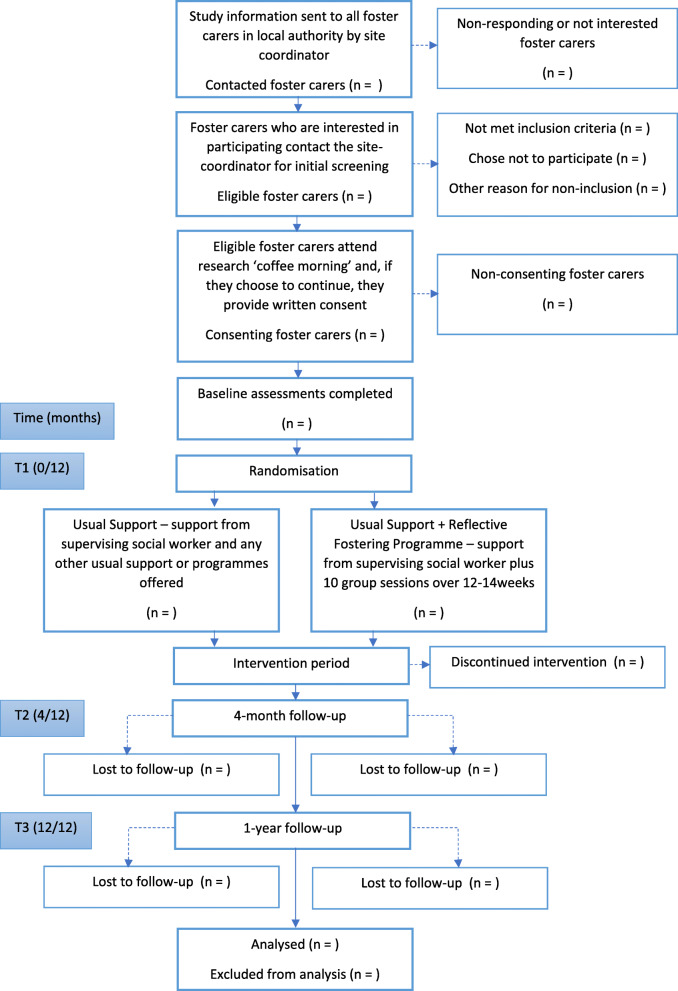
CONSORT diagram

A CONSORT diagram can be seen below (Fig. 1).

After providing consent, participants will be asked to complete the foster carer demographic form and study outcome measures before they are randomly allocated to either the Reflective Fostering Programme (intervention) or the control group. Those participants unable to complete the study measures online will be able to complete them with a member of the research team by telephone. If they cannot be completed either online or by telephone, participants will be provided with paper copies to complete and return (if COVID-19 restrictions allow).

Participants allocated to the intervention arm will begin to attend the Reflective Fostering Programme and those in the control arm will continue with their usual social care support. The intervention period will last between 12 and 14 weeks.

Participants will be asked to complete the same study outcome measures as at baseline at two time-points: 4 months (± 4 weeks) and 12 months (± 4 weeks) after baseline. They will be contacted by the research team by text, email, or phone call and asked to complete the assessments. Those participants unable to complete the study measures online will complete these with a member of the research team by telephone. If they cannot be completed either online or by telephone, participants will be provided with paper copies to complete and return (if COVID-19 restrictions allow).

If the child is no longer with the same carer at either the 4-month or 12-month follow-up, relevant follow-up assessments will still be completed with the original foster carer. Where possible, the child’s current carer will be contacted by the local authority to seek their consent to be contacted by the research team. If they agree to be contacted, a member of the research team will seek consent for the new carer to complete the child-focused measures in relation to the original ‘nominated’ child. The feasibility of doing this has already been established in the Herts and Minds study [48]. The Site Lead, or their delegate, will be responsible for identifying the current carer and approaching them to provide consent to complete study outcome measures.

See Table 1. below for the study schedule.

**Table 1:** Study schedule

	Study period						
	Screening	Randomisation	Post-randomisation				Process evaluation
Timepoint	Pre-T0	Pre-T0	T0 Baseline	T0–T1	T1 + 4 months	T2 + 12 months	
**Enrolment:**							
**Eligibility screen**	X						
**Coffee Morning**	X						
**Informed consent**	X						
**Randomisation**		X					
***Fostering Team profile questionnaire***	X						X
**Delivery of Reflective Fostering Programme**				X			
**Attendance Log**				X			
**Facilitator Adherence Rating**				X			
**Incidents of concern Log**				X			
**Assessments:**							
***Demographic information***			X				
***Strengths and difficulties questionnaire***			X		X	X	
***Parenting Stress Index – short form***			X		X	X	
***Professional QoL questionnaire***			X		X	X	
***Emotion Regulation Checklist***			X		X	X	
***Carer Defined Problem Scale***			X		X	X	
***Child Health Utility-9D***			X		X	X	
***Child and Adolescent Service Use Schedule***			X		X	X	
***Placement Stability***			X		X	X	
***Focus Groups with facilitators***							X
***Interviews with foster carers***						X	

### Sample size {14}

Previous experience with this population [39] suggests an effect size ranging from *d* = 0.3 to *d* = 0.4 (i.e. a mean difference in outcome between groups of 0.3 to 0.4 standard deviations) to be clinically relevant. In the first feasibility and evaluation study [28], the baseline standard deviation for the primary outcome measure (SDQ) was 6.8 and showed a change equivalent to an effect size of *d* = 0.3. The current trial compares usual support plus the Reflective Fostering Programme to usual support alone, with the primary outcome assessed at 12 months. We have, therefore, based our sample size on a difference of 0.3 standard deviations in the SDQ, i.e. around 2.0 units.

On the basis of a review of previous clinical trials involving parenting programmes for foster carers, a reasonable estimate for dropout rates for the study is in the range is of 10–15% at the end of intervention (4 months), and 20–25% at 12 months.

The trial is randomised at the individual participant level. However, the nature of the intervention in group format will imply a form of ‘clustering’, i.e. outcomes amongst those in the same intervention groups may be correlated; the strength of this correlation is quantified through an intra-class correlation co-efficient (ICC). Whilst the intervention for the control group has a less clear ‘clustered’ structure, as usual care will vary according to each site’s usual practice, the assumption of zero clustering is likely to be incorrect, due to the delivery by allocated social workers. Therefore, we will consider both arms to have the same degree of ‘clustering’ to avoid potentially under-estimating the sample.

Table 2 provides the estimated statistical power from four different sample sizes across eight different scenarios (varying by assumed ICC and drop-out rate). These estimates assume an average of seven foster carers in each intervention group, an effect size of 0.3 and a fixed statistical significance of 5% (two-sided). A target sample size of 720, 360 per arm, has been fixed for this trial. This was based upon the likelihood of a statistical power between 80 and 90% in most reasonable scenarios.

**Table 2:** Statistical power estimates for differing drop-out rates and ICCs

	Drop-out	15%		20%		25%		30%	
	Assumed ICC	0.05	0.10	0.05	0.10	0.05	0.10	0.05	0.10
	770	93%	88%	91%	86%	90%	85%	88%	82%
Total	720	91%	86%	90%	85%	88%	83%	83%	77%
Randomised									
	620	88%	83%	86%	81%	84%	79%	87%	76%
	500	82%	77%	79%	75%	78%	73%	82%	71%

### Recruitment {15}

Recruitment of foster carers will take place across five cycles of recruitment and delivery (including the pilot phase), timed in such a way that the start of each new wave of Reflective Fostering Programme groups will begin close to the start of a school term (i.e. January, April, and September).

The research team will recruit additional local authorities if recruitment during the first two waves is slower than anticipated (e.g. due to COVID-19 restrictions). This will ensure sufficient numbers of foster carers are recruited to the study. The study team will work with partners including Research in Practice, the Social Care Learning Network, and the Clinical Research Network (CRN) to identify suitable sites. Selection of sites will take into account the geographic and cultural diversity of carers and children in care in the UK, including both more rural and metropolitan settings, in order to ensure that both the intervention and the research study meet the needs of a culturally diverse population living in different parts of the country, and that any obstacles or barriers are identified as part of the process evaluation.

Foster carers will be recruited from local authority sites. Information about the study will be disseminated to foster carers primarily through local authority fostering support teams, but also through local foster carer support groups and by supervising social care workers. All foster carers will be sent information about the study via their local fostering support teams. Foster carers will be encouraged to consult with their supervising social worker about their suitability for the study. If interested, foster carers will be asked to contact the Site Coordinator by phone or email to register their interest in finding out more about the study.

Only one foster carer from each household can join the study as a participant. However, it is recognised that some foster carers randomised to the intervention arm may want to attend the Reflective Fostering Programme together. For those participants, there will be the option for a second carer from the same household to attend together, if they wish to do so. The attendance of both will be recorded, but only the primary carer will be asked to complete study outcome measures. We will report on how many couples choose to attend.

Foster carer recruitment targets for each site will be proportionate to overall size, with the aim to recruit more than 50% of all eligible carers over a 20-month recruitment period in order to meet recruitment targets. This is in line with previous clinical trials involving training programmes for foster carers, where 58–62% of eligible carers are reported to have participated [41]. However, should recruitment be lower than anticipated, potential barriers to dissemination and recruitment will be examined, and if needed, additional local authorities may be recruited.

## Assignment of interventions: allocation

### Sequence generation {16a}

Randomisation will be allocated in a 1:1 ratio, stratified by age of child (4–9 vs 10–13), number of previous placements (1 or less, vs 2 or more) and recruiting region. Consenting foster carers will be randomly allocated to the intervention arm (Reflective Fostering Programme in addition to Usual support) or the control arm (Usual support). The randomisation sequence will be computer generated, blocked in random block lengths of two or four.

### Concealment mechanism {16b}

The allocation sequence will be implemented using randomisation via the REDcap database. Only the database administrator will be permitted to view the allocation sequence and therefore the sequence will be concealed prior to intervention allocation.

Participants will be informed of group allocation by the local Site Coordinator, who will also contact the relevant social worker/s, so that they are aware that the foster carer is taking part in the study. If they are in the intervention group, the Reflective Fostering Programme facilitators at each site will be informed.

### Implementation {16c}

Participants will be randomised as soon as they complete baseline measures. The allocation sequence will be generated by Norwich Clinical Trials Unit (NCTU) Data Management. Randomisation will be managed online (using a tool built by NCTU Data Management) and overseen by the TM.

## Assignment of interventions: blinding

### Who will be blinded {17a}

Both outcome assessors and the trial statistician will be blinded after assignment to interventions. The participants may require the help of members of the research team to help them complete study measures. If so, participants will be reminded not to reveal which arm they are in.

Due to the nature of the intervention, it is not possible for facilitators or foster carers to be blind to allocation. The Site Coordinator will be unblinded so that they can coordinate and plan the Programme delivery. Access to the database will be defined for each role in the study to maintain blinding. The Trial Manager (TM) will also be unblinded to enable any queries from participants relating to the research to be addressed. Within the REDCap study database, the blinded or unblinded status of users will be preserved by database permissions and defensive programming.

### Procedure for unblinding if needed {17b}

The Trial Steering Committee (TSC) is responsible for reviewing safety events and may request that unblinded data from the database be made available to an independent group, should there be concerns raised by the TSC with respect to the number of events and whether this is associated with the intervention.

## Data collection and management

### Plans for assessment and collection of outcomes {18a}

#### Data collection process

All instruments consist of online-administered self-report questionnaires which can be accessed remotely on the foster carers’ own devices (computer, phone, or tablet). A researcher that is blind to study arm will be available to assist with completion of self-report measures by telephone, if required. In this case, participants will be asked not to disclose their group allocation to the researcher. In exceptional cases, and subject to ethical approval in light of COVID-19 restrictions, participants who would rather complete the measures offline, in private and at their own pace will be able to complete paper versions of measures to be returned to a member of the research team who will enter it onto the database (if COVID-19 restrictions allow).

Data will be collected at baseline (immediately prior to the intervention), 4 months post-baseline, and 12 months post-baseline. It is anticipated that a significant proportion (around 70%) of identified children will be with the same foster carer at both the 4- and 12-month follow-up points [42]. Although it is possible that placements may have changed since the initial programme, we have confidence that follow-up data can be collected in the majority of cases even if the nominated child has moved to a new placement. Previous experience has established that it is possible to successfully seek consent from new foster carers to provide follow-up data [45].

Participants will be asked to complete the foster carer demographic form and study baseline measures before randomisation takes place.

### Data collection instruments / outcome measures

Unless otherwise indicated, all study measures have established validity and reliability, and are widely available. Specific study measures are available from the corresponding author.

#### Primary outcome measure

Child emotional and behavioural wellbeing will be measured by the *Strengths and Difficulties Questionnaire* (SDQ [33];), which is a routinely used clinical tool completed by caregivers and designed to assess emotional and behavioural difficulties in children aged between 3 and 17 years. It was used in the preliminary evaluation and feasibility study [31] and is recognised as the UK government’s preferred measure of wellbeing for children in care. The SDQ consists of 25 closed-ended questions and an impact supplement, which assesses the extent to which mental health problems have had an impact on aspects of the child’s life. Each item is rated using response categories of ‘Not-true’, ‘Somewhat true’, and ‘Certainly true’. The 25 items are scored across five (five-item) subscales: *Conduct problems, Emotional problems, Hyperactivity, Peer problems,* and *Prosocial.* For all the subscales, apart from the prosocial subscale, higher scores are designed to indicate that there are greater levels of difficulty. A total difficulties scale is generated by summing all of the scales except for the prosocial scale.

#### Secondary outcome measures

Parental stress will be measured using the *Parenting Stress Index – Short Form* (PSI 4-SF; [34]), which is a self-report measure comprising 36 items using a 5-point Likert scale; designed to assess perceived stress in the parenting role. The 36 items are divided into three 12-item subscales; Parental distress, Parent-child dysfunctional interaction, and Difficult child, which combine to form a total perceived stress score. Higher scores on these scales, and on the total stress score, are meant to reflect greater difficulties, with a score of 90 and above (on both the total score and subscales) falling within the ‘clinical’ range.

*The Parental Reflective Functioning Questionnaire* (PRFQ; [37]) will be used to measure the carers’ capacity for reflective functioning in their caregiving role. The PRFQ is an 18-item questionnaire including three main subscales: *Pre-mentalizing,* which assesses non-mentalizing of the child, or inability of the parent to acknowledge their child’s mental states; *Certainty about mental states*, which measures how certain caregivers are about the mental states of their child and their ability/inability to recognise the opacity of mental states; and *Interest and Curiosity*, which is designed to measure parental interest and curiosity in their child’s mental states.

High scores in Pre-mentalizing and Certainty about mental states reflect reduced capacity for reflective functioning, and high scores in Interest and Curiosity represent increased reflective functioning.

*The Professional Quality of Life Questionnaire* (PRoQOL) [35] will be used to measure foster carer compassion fatigue and burnout. The PRoQOL is a 30-item self-report measure of the positive and negative effects of working with people who have experienced extremely stressful events. Items are scored using a Likert-type scale where 1 = Never and 5 = very often. The PRoQOL contains three subscales measuring Compassion Fatigue, Burnout and Compassion Satisfaction.

*The Emotion Regulation Checklist* (ERC; [36]) will be used to assess the carer’s view of a child’s emotions. The ERC consists of 24 items scored using a 4-point Likert-type scale, where 1 = Never and 4 = Always, designed to be completed by an adult who knows the child well, such as a parent or carer. The measure has two subscales: *Lability/Negativity* (15 items), which is designed to assess emotional intensity, expression of negative emotions, arousal and reactivity, and lability of mood, and *Emotion regulation* (8 items), which measures adaptive regulation, such as socially appropriate displays of emotion, empathy and emotional understanding. Higher scores on the *Lability/Negativity* scale reflect greater dysregulation, whilst higher scores on the *Emotion regulation* scale indicate a higher capacity for emotion regulation.

*Carer-Defined Problems Scale* (CDPS; [38]) is a measure adapted from the Goal-Based Outcome Measure (GBO; [43, 44]). The measure asks carers to rate and record up to three problems at the beginning of the intervention (‘Please List below, in order of priority, three problems you have with your child that you would most like help with. Then rate the severity of the problem at present by indicating a number from 0 to 10’). In this version, adapted for online use, changes on the scale are rated by participants on a scale from 0 (no longer a problem) to 10 (couldn’t be worse). The outcome is the amount of movement along the scale from the start to the end of the intervention. Evidence from Briskman and colleagues (2012) has indicated that this measure is highly sensitive to change for foster carers reporting on identified problems with the ‘target’ children they are considering in relation to a parenting-based intervention [45].

The *Placement Stability Log* has been developed for use in this study and will be used to collect data relating to changes of social worker, change of school or placement change and reasons for any change. The Placement Stability Log asks carers to report when there has been any significant events or changes relating to the child’s placement. It asks about any changes to the child’s foster placement and if so, where to and the reasons why. Lastly it asks about foster child-related events.

Data from this measure will be used to track placement stability and inform collection of follow-up measures, e.g. if a child has moved placement.

#### Economic evaluation measures

##### Child Health Utility 9 Dimensions (CHU9D) [40]

A paediatric generic, preference-based measure of health-related quality of life, to be completed by the carer. The CHU9D covers nine dimensions (worried, sad, annoyed, tired, pain, sleep, daily routine, work, able to join in activities), each rated on five levels, and can be used to generate quality-adjusted life years (QALYs) for use in cost-utility analysis.

##### Child and Adolescent Service Use Schedule (CA-SUS)

Service-use data will be collected using an adapted version of the CA-SUS, a measure originally designed for young people in mental health populations, but which has been adapted and successfully implemented in a range of health and social care-based studies [46, 47]. The version we will use was designed and tested for use with children in care in an earlier feasibility study [39, 48] and an online, self-report version is now being developed. The CA-SUS will be completed remotely by carers at baseline, covering the previous 3 months, and at both follow-ups (4- and 12-months post-baseline), covering the period since last assessment. The CA-SUS will be reviewed at the end of the Pilot Phase and has been adapted to ensure coverage of remote online and telephone delivery of services, given the changes to service delivery as a result of Covid-19 restrictions.

#### Other measures

##### Foster carer demographics form

Basic demographic information about foster carers will be collected at baseline using the *Foster carers demographics form*. This 15-item questionnaire will ask foster carers to provide information about themselves (e.g. Age, gender, ethnic background, educational history and marital status), their fostering history, and demographic and fostering history of the child in their care.

##### Fostering team site profile questionnaire (SPQ)

Service characteristics of local authorities will be collected at the beginning and end of the trial period via a *Fostering team site profile questionnaire*. This 16-item questionnaire will gather information which characterises services, including numbers of children placed in foster care, numbers of registered foster carers, and associated interpreting services, and existing foster caring policies, training, and support programmes in place. Data from this measure will allow researchers to identify changes in the service between pre- and post-intervention.

#### Facilitator evaluation

As part of the embedded process evaluation (see below), facilitator adherence will be measured using the Reflective Fostering Programme *Facilitator Adherence Rating (FAR)* (Unpublished tool): a 14-item observation-based assessment, covering the key components of the Reflective Fostering Programme, used to assess facilitators’ adherence to the Programme.

#### Interview schedule (Foster carer interviews)

An interview schedule will be used to guide semi-structured interviews with foster carers as part of the process evaluation. The interview schedule will include the following topics:

- Experience of being a foster carer
- Experience of receiving the intervention/usual support
- The Reflective Fostering Programme (Intervention arm only)
- The type of fostering support received (control arm only)
- Wider experiences of taking part in the study

Topics and discussions may vary slightly depending on what group the foster carer was allocated to. Interviews are expected to take no longer than 1 h.

#### Topic guide (focus groups with facilitators)

Focus groups with facilitators will take place to understand experiences of programme delivery, including co-delivery by carers and social workers, and how the wider context of local authorities influenced intervention delivery. A topic guide has been developed to elicit conversation and ensure all essential topics are covered in the focus group. Topics to be covered include:

- Facilitator experiences of delivering the Programme
- Usefulness of the Reflective Fostering Programme training
- The Reflective Fostering Programme
- Experiences of co-delivery
- Weekly supervision
- Wider experiences of taking part in The Reflective Fostering Study

Focus groups are expected to take no longer than 1.5 h.

### Plans to promote participant retention and complete follow-up {18b}

All participants will be provided with a unique username and password so they can sign into the database and complete the study measures. Foster carers will be automatically reminded by text, email, or phone call to complete the assessments by the research team, and will be followed up in the same way, if necessary, to encourage completion. Those participants unable to complete the study measures online will complete these with a member of the research team by telephone. If they cannot be completed by telephone, participants will be provided with paper copies to complete and return (if COVID-19 restrictions allow). This will ensure that participants who might not be confident in using technology will still be able to complete the measures.

Participants who withdraw from the intervention will be asked to continue to provide outcome data. For participants who discontinue with the study itself, a study withdrawal form will be completed detailing how the participant communicated their wishes to withdraw, type of withdrawal, date, reasons (if obtained), and any discussion regarding withdrawal.

### Data management {19}

Data will be entered under each participant’s Participant ID (PID) onto the central database, stored on servers based at NCTU by members of the study team working within each research site, and collected at the time-points indicated in the Trial Schedule. Randomisation of participants will also be implemented within this database.

Data collection, data entry, and queries raised by a member of the study team will be conducted in line with the NCTU and study-specific data management processes. Identification logs, screening logs, and enrolment logs will be kept at the study site in a locked cabinet within a secured room. Electronic copies of these logs will be kept on the Investigator Site Files which will be password protected. All data will be handled in accordance with the Data Protection Act 2018.

Access to the database will be via unique, individually assigned (i.e. not generic) usernames and passwords. The database will be accessible only to members of the study team, and external regulators if requested. Functional access within the database will be controlled and limited by role and, where appropriate, by site. This access to the study database is controlled and administered by NCTU Data Management. The servers are protected by University of East Anglia (UEA) firewalls and anti-virus products and are patched and maintained (including back-ups) according to best practice. The physical location of the servers is environmentally controlled and protected by CCTV and security door access.

Participant identifiable data will be stored in the database to enable participants to be contacted by site staff for the purpose of sending questionnaires. There will be a clear logical separation of participant identifiable data from the trial data (i.e. by user/role permissions and by data collection instrument).

The database software provides a number of features to help maintain data quality, including maintaining an audit trail, allowing custom validations on all data, allowing users to raise data query requests, and search facilities to identify validation failure/ missing data.

After completion of the study, the database will be retained on the servers of NCTU for on-going analysis of secondary outcomes. The study database and associated design documentation will be routinely archived for a period of 5 years unless otherwise advised by the Trial Management Group (TMG). Note that all identifying information such as email addresses will be removed prior to archiving.

### Confidentiality {27}

Confidentiality and anonymity will be ensured throughout the study. For those in the intervention arm, participants may discuss sensitive matters during recorded sessions, and any participants may discuss sensitive matters during interviews or focus groups. This will be managed through close attention to confidentiality. The reporting of results (including quotations) will be fully anonymised and excerpts will only be used with the explicit consent of participants.

The study staff will ensure that the participants’ anonymity is maintained. The participants will be identified only by a Participant ID number on all study documents and any electronic database. Additionally, participant identifiers required for automated communication (including email address and mobile telephone number) will also be stored in the database, but logically separated from study data by interface and permission constraints. All documents will be stored securely and only accessible by study staff and authorised personnel. The study will comply with the Data Protection Act 2018, which requires data to be anonymised as soon as it is practical to do so.

Following the Caldicott Principles, only data to be used in analysis relevant to the study and to facilitate running the study will be collected, limited to the surveys and demographic material to be used for data analysis, and contact details for electronic communication. All participants will be identified by a unique ID code that will only be linkable to their name via a password-encrypted excel file. Only a limited number of researchers will have access to the link between participant names and ID numbers.

An electronic CRF will be produced. Each participant will have a corresponding CRFs unique to them. CRFs will not bear the participant’s name. The participant’s initials, date of birth, and study PID will be used for identification on the database. Access to the database will be managed by NCTU and will be restricted and controlled to authorised personnel and will be password protected. The audit trail will be monitored regularly for any unauthorised access. It is the responsibility of the CI/Site Lead(s) to ensure that relevant personnel are delegated to carry out data collection and data entry. The delegation log will identify all those personnel with responsibilities for data collection and handling, including those who have access to the trial database.

### Plans for collection, laboratory evaluation, and storage of biological specimens for genetic or molecular analysis in this trial/future use {33}

Not applicable, no samples collected.

## Statistical methods

### Statistical methods for primary and secondary outcomes {20a}

Data collected from foster carers who were approached but chose not to participate and those who withdrew during the study will be analysed for any trends. All data collected up to the point of withdrawal of consent will be retained in the study dataset and will be included in the analysis according to the intention-to-treat principle.

#### Primary analysis

A general linear model, using general estimating equations (GEE), will be used for the analysis of the primary outcome, carer-reported SDQ at 12 months. The GEE approach to estimation will incorporate the clustering in outcome values by intervention groups within the intervention arm. As there is no overt clustering in the control arm (i.e. an example of ‘partial clustering’), each participant within the control can be considered a ‘cluster of one’ for analysis purposes. The linear model will include recruiting ‘site’ (as a random effect), the SDQ score at baseline, age, and previous number of placements (design factors) and intervention group. The primary outcome, SDQ, is assumed to follow a normal distribution. The residuals from this model will be checked against this assumption. The primary analysis will use the intention-to-treat principle, i.e. analysing participants according to the group to which they were randomly allocated, irrespective of intervention received.

#### Secondary outcome analysis

Secondary outcomes, including the SDQ at 4 months, will be analysed in a similar fashion, i.e. through a linear model using GEE with an appropriate link and error term depending upon the nature of the outcome. No subgroup analyses are currently planned, but should the need for subgroup analyses become apparent during the course of the study (for example due to new information), these will be specified in the Statistical Analysis Plan (SAP) and most likely analysed using an interaction effect in the linear model. If the study includes a mix of face to face and online delivery of the Reflective Fostering Programme, a formal subgroup analysis comparing the two will be applied. This will involve the addition of a group-by-delivery type intervention term. However, the study has not been primarily designed to address any differences in outcome associated with delivery format.

Reflective capacity, as measured by the PRFQ, is a target of the intervention and, therefore, needs to be considered as one of the secondary outcomes. However, reflective capacity is also one of the mechanisms of change of the intervention and may be important in mediating the effect of the intervention on both the child and the foster carer. Reflective capacity will therefore be considered as both a secondary outcome and a mediating factor.

### Interim analyses {21b}

There are no planned formal interim analyses. Summaries of safety and efficacy data will be considered on a regular basis by the oversight committees.

### Methods for additional analyses (e.g. subgroup analyses) {20b}

Three potential moderators of outcome have been identified and a formal subgroup analysis will be undertaken corresponding to each. The three are (1) the foster carers reflective caring at baseline; (2) the child’s age (from 4 to 9, versus 10 to 13); and (3) the number of previous placements (none or one, versus 2 or more). In each case, the analytical model for efficacy described above will have to be extended to include an interaction term, i.e. the interaction between subgrouping variable and treatment arm. No formal sample size calculation has been carried out and the study has not been designed to provide a set level of statistical power for these subgroup analyses. Additional subgroup analyses may be suggested during the course of the study in the light of emerging information from other studies. These will be kept to a minimum and explicitly stated in the SAP.

The change in foster carer’s parental reflective functioning is proposed as a mediator of outcome, i.e. an intermediary variable on the causal pathway between intervention and primary outcome. To test this, a formal mediation analysis will be carried out using the approach suggested by Baron and Kenny [49]. This involves firstly modelling the relationship between intervention and parental reflective functioning and between parental reflective functioning and outcome (i.e. along the mediated route), plus modelling the relationship between intervention and outcome (i.e. the direct route) whilst adjusting for parental reflective functioning. This analysis will only be carried out, conditional on a significant effect of intervention being found in the primary analysis.

#### Data analysis for programme fidelity

All sessions of the Reflective Fostering Programme will be video-recorded and programme fidelity will be assessed using the Reflective Fostering Programme FAR. Four 5-min sections from each session will be purposively sampled and rated by one of the team of consultants involved in providing consultations to facilitators. To establish inter-rater reliability, consultants will be provided with training in the FAR prior to beginning rating and their inter-rater reliability will be assessed. Treatment fidelity will be considered satisfactory if sessions are rated with a mean score of 3 or above (‘adequate’) across the 14 items.

### Methods in analysis to handle protocol non-adherence and any statistical methods to handle missing data {20c}

The primary analysis will be on an intention to treat analysis, i.e. individuals will be analysed according to the group to which they were randomised irrespective of the intervention actually received. No individual will be removed from the analysis on accordance of non-adherence to the treatment protocol. Post-randomisation exclusions (PREs), i.e. those later discovered not to be eligible for the study, will not be included in any data analyses.

#### Missing data

Missing data will be considered with potential mechanisms. If missing data is less than half but more than a trivial amount (say, 2%), then multiple imputation will be used with an imputation model containing at least those variables in the analytic model.

### Plans to give access to the full protocol, participant level-data, and statistical code {31c}

The full study protocol is available on the NIHR website: https://www.fundingawards.nihr.ac.uk/award/NIHR127422

Requests for access to the data should be made to the CI and/or Sponsor (University of Hertfordshire).

## Additional phases

### Internal pilot phase

An internal pilot phase will run for the first 6 months and has been designed to allow an assessment of stop/go criteria before committing to the remainder of the study. During the internal pilot study, the aim will be to recruit sufficient participants, across four sites, with each site running one Reflective Fostering Programme group (6–10 participants per group) and one control group of foster carers (matched number of participants).

To explore any challenges identified in the recruitment and randomisation procedures, we will review data from the ‘non-participation’ questionnaire. Data from those eligible carers who chose not to participate will be analysed to identify any systematic obstacles to participation and reasons for not agreeing to take part. We will also hold telephone or online interviews with carers (10 per arm) at 4 months from baseline in order to identify carer’s perspectives of how the Reflective Fostering intervention is received, and in the control arm to assess any contamination.

A 1-day ‘feedback and problem-solving’ workshop will be held towards the end of the internal pilot to identify challenges of recruitment, randomisation, retention, contamination, and delivery of the Reflective Fostering intervention. Participants will include the research team, Reflective Fostering Programme facilitators, service managers, and administrators responsible for screening potential participants. During the workshop, we will hold group discussions to elicit feedback from different professionals working across the pilot sites in order to identify problems and solutions, which we can use to improve delivery of the study.

At the end of this phase, a decision will be made by the funder, in consultation with the TSC on whether to continue with the study. Recruitment will continue whilst data on patients in the internal pilot are analysed and reviewed by the TSC and a funder decision is obtained. As an internal pilot, all data collected on study participants will be included in the full study analyses.

The objectives of the internal pilot phase are to confirm feasibility of:

1. Foster carer recruitment
2. Foster carer retention
3. Intervention and outcome assessment delivery, including the feasibility of online delivery, if COVID-19-related restrictions mean that face to face delivery is not possible

The stop/go criteria are:

1. Participant (foster carer) recruitment at expected rate (more than 50% of eligible participants), or evidence that identified barriers to recruitment can be overcome
2. Evidence of retention in the study at 4 months (less than 15% drop-out from the study), or evidence that identified barriers to participant retention can be overcome.
3. No clear evidence of contamination across arms that cannot be remedied.
4. Evidence that the interventions and training can be delivered, whether remotely or face to face, and assessments can be completed within the proposed timeframes for the definitive study, or evidence that identified barriers can be overcome.

Participants who discontinue with the Reflective Fostering Programme will remain in the study for the purpose of data collection, follow-up, and data analysis unless explicitly stated by the participant. All data collected up to the point of withdrawal of consent will be retained in the trial dataset. Participants who agree to complete follow-up data should continue to be followed up as closely as possible to the follow-up schedule defined in the protocol, providing they are willing and are still able to consent to this. If, however, the participant exercises the view that they no longer wish to be followed up, this view must be respected, and the participant withdrawn entirely from the study.

Data already collected will be retained and included in analyses according to the intention to treat principle for all participants who withdraw consent. This will be made clear within the information sheet.

### Economic evaluation

The aim of the economic evaluation is to establish whether the provision of the Reflective Fostering Programme in addition to usual support would be a cost-effective and worthwhile use of local authority fostering team’s resources, compared to usual support alone. The economic evaluation will take a broad perspective covering (a) the NHS/personal social services perspective preferred by NICE, including any education-based health or social care services, given the age of the population, as well as health and social services provided by the private or non-statutory sectors and (b) education facilities, to capture any use of specialist schools. We will include all health and social care services, not just those directly related to the intervention.

The Reflective Fostering intervention will be directly costed using a micro-costing (bottom-up) approach [50]. Data on the Reflective Fostering Programme groups, including attendance, will be collected from facilitators. The salary costs of the group facilitators including employer on-costs (national insurance and superannuation) and appropriate overheads (capital, management, administration, etc.) will be weighted to include relevant non-face-to-face time spent on other activities (e.g. session preparation, writing up notes, meetings, training) and used to calculate a cost per group. Cost per group will then be allocated across all foster carers invited to attend on the basis that the workshops are closed groups and will go ahead irrespective of attendance [51]. All other services will be costed using nationally applicable unit costs (e.g. Personal Social Services Research Unit (PSSRU) Unit Costs of Health and Social Care compendium, NHS Reference Costs for hospital contacts, British National Formulary for medications).

The primary economic evaluation will be a cost-effectiveness analysis carried out at 12 months post-randomisation (T3) with outcomes expressed in terms of the primary measure of outcome (SDQ), in line with the primary clinical research question. Although cost-utility analysis using quality-adjusted life years (QALY) is preferred by NICE, measures for the estimation of QALYs are health-related measures of quality of life (QoL), which may be too narrow to reflect the broader impact of the proposed intervention on the QoL of the young participants. Although NICE also recommends the use of capability or social care-related quality of life measures where an intervention results in both health and capability or social care outcomes [52], there is currently no capability or social care measure capable of generating QALYs which is suitable for children.

Instead, a secondary economic evaluation will explore cost-utility using QALYs generated from the CHU9D measure of health-related quality of life [40], proxy reported by foster carers. Guidance for proxy report and for application in children under the age of 7, available from the developers, will be adhered to. QALYs from the CHU9D will be calculated using the recommended area under the curve approach [53]. The CHU9D was selected because it covers a wide range of dimensions (nine dimensions: being worried, sad, annoyed, tired, in pain, sleep, daily routine, school work, and usual activities), which is broader than measures such as the EQ-5D-Y [54], the youth version of the EuroQol-5 dimensions measure of health-related quality of life, or the Pediatric Quality of Life Inventory (PedsQL) [55], a broader measure of quality of life but still with a narrow range of dimensions.

#### Data analysis for health economic analysis

Costs and outcomes will be compared in terms of mean differences and 95% confidence intervals from non-parametric bootstrap regressions (1000 replications) to account for non-normal distribution common to economic data. Missing data will be imputed using multiple imputation using chained equations [56], and all analyses will be adjusted for covariates in line with the data analyses for the main study (described above). Cost-effectiveness will be assessed using the net benefit approach following standard approaches [57]. A joint distribution of incremental mean costs and effects for the two groups will be generated using non-parametric bootstrapping to explore the probability that each of the treatments is the optimal choice, subject to a range of possible maximum values (ceiling ratio) that a decision-maker might be willing to pay for improvements in outcome (SDQ and QALYs). Cost-effectiveness will be explored using incremental cost-effectiveness ratios [58], with uncertainty represented by cost-effectiveness planes and cost-effectiveness acceptability curves [59].

With regard to reference costs compared to other clinically trialled programmes designed to assist foster carers, we will explore the availability of cost-effectiveness evidence for other interventions that may be available for foster carers, to support such comparisons. Review of available evidence will be carried out close to the end of the study to ensure that we identify any new studies completed and published during the course of the study.

### Process evaluation

During the main RCT, a parallel mixed methods process evaluation will:

- Characterise ‘usual support’,
- Investigate how different service models and the wider context of local authorities’ shapes intervention delivery,
- Evaluate implementation and theoretical fidelity to the Reflective Fostering intervention,
- Evaluate how intervention implementation is affected by face to face, online or blended delivery,
- Identify how carers experienced the intervention,
- Assess non-receipt of the intervention in the control arm,
- Provide explanations for the observed effects in the main study, and
- Identify strategies for wider implementation of the Reflective Fostering intervention.

Using the SPQ, all local authorities will be characterised at the beginning and end of the study period to identify service characteristics (i.e. numbers of children placed in foster care, numbers of registered foster carers, and associated interpreting services, and existing foster caring policies, training and support programmes in place), and changes in the service which might affect implementation of the Reflective Fostering intervention throughout the duration of the study.

Findings from the SPQ will be used to purposively select four case study sites to obtain maximum variation in local authority characteristics. In these sites only, the training of facilitators will be observed to understand how the principles and content of the Reflective Fostering intervention is transferred to Reflective Fostering Programme facilitators. If the intervention is delivered using a mixture of face to face and online delivery then a key consideration will be how implementation is affected by these different modes of delivery. A sub-sample of session recordings (approx. 5 h) will be purposively selected, based on FAR ratings, to further understand how the principles of the Programme are enacted between facilitators and carers. Extracts from sessions will initially be selected to include sections where the theoretical mechanisms underpinning the Reflective Fostering intervention (i.e. mentalisation, reflective capacity, and enhanced monitoring one’s ‘emotional temperature’) are intended to be delivered (see Theory of Change model in Appendix 1). Extracts will be transcribed verbatim and using a Conversation Analytic approach [60, 61], verbal and non-verbal communication will be analysed for evidence of how the theoretical mechanisms are enacted by facilitators and received by carers within sessions.

Face to face, online, or telephone interviews in the four case study sites will coincide with the 12-month (T3) follow-up assessment. Consent to be contacted about the interviews will have been sought at the information (coffee morning) event, where the process evaluation element will be fully explained to potential participants. Details of those who have provided optional consent to be contacted to take part in the interviews will be included in the study database and be available to the process evaluation team. Those participants who have given consent and are purposively selected (see below) will be provided with information about the interviews/focus groups and asked to provide consent to take part. Face to face, online, or telephone interviews with purposively selected carers in the intervention arm (five per site across the four case study sites) will take place. Carers will be purposively sampled to obtain maximum variation across years of experience as a carer, whether caring for children aged 4–9 years or 10–13 years, whether a kinship or foster carer, and whether one or both carers receive the intervention. This will be to understand their experience of receiving the intervention, how it affected their care of children, the extent to which this was sustained after the training programme was completed, and the source of any other support obtained during the study. Face to face, online, or telephone interviews with 20 purposively selected carers in the control arm (5 per site) will be conducted to assess non-receipt of the intervention and experience of usual support, sampled to match characteristics of those interviewed in the intervention arm. Interviews will be guided by a semi-structured interview schedule, which will include key topics to explore (see section ‘Plans for assessment and collection of outcomes {18a}’ for details of the semi-structured interview schedule).

Face to face or online focus groups with those delivering the Reflective Fostering Programme in the four case study sites will take place to understand experiences of programme delivery, including co-delivery by carers and social workers, and how the wider context of local authorities influenced intervention delivery. With the permission of participants, semi-structured interviews, focus groups, and observations of intervention delivery will be (audio/video) recorded and transcribed verbatim. Audio data will be uploaded onto OneDrive and filed using participant IDs before removing from the recorder. Data will be transcribed by a professional transcription company who will be required to sign a confidentiality agreement and all identifiable information will be removed from the transcripts.

All interviews, focus groups, and observations of intervention delivery will be analysed using NVivo software. In the intervention arm, we will then develop a coding scheme to thematically analyse how the process and content of the Reflective Fostering intervention functioned from the carer’s perspective. A constant comparison approach will be adopted, working iteratively between data obtained from different interviewees within and between local authorities.

#### Process evaluation data synthesis

The analysis of process evaluation data will be iterative, moving between data collection and data analysis to test emerging theories. It may, for example, emerge that some carers have expectations about Reflective Fostering, which shape their experience and use of the intervention, and this may require deeper exploration. The analysis of the video/audio-recorded sessions will therefore require knowledge from carer interviews to compare how reported experience relates to actual implementation of the intervention. Care will be taken to identify and follow up deviant cases which do not fit into emerging theories. This approach will involve working laterally across data types, focusing on identifying ‘telling cases’, triangulating and looking for connections between data. Emerging theories and the relationship of the data to the conceptual literature underpinning the intervention will be discussed and refined at team meetings throughout the research.

By examining the delivery of the Reflective Fostering intervention within the wider context of the local authorities, we will be able to make the transition from the identification of routines and patterns of use of the intervention in specific sites, to theoretical explanations of how different structural relations organise different moments of delivery, which then impact on the specific outcomes we observed in the main study findings. In doing so, we will be able to identify factors plausibly and/or consistently related to successful or unsuccessful delivery of the intervention, enabling the generation of strategies for wide-scale implementation of the Reflective Fostering intervention.

## Oversight and monitoring

### Composition of the coordinating centre and trial steering committee {5d}

#### Trial Steering Committee (TSC)

The TSC will meet twice a year and will provide overall supervision for the study on behalf of the study Sponsor and the Funder. It will ensure that the study is conducted to the rigorous standards set out in the Department of Health and Social Care’s (DHSC) Research Governance Framework for Health and Social Care and the Guidelines for GCP. The TSC will operate according to NIHR Evaluation Trials and Studies Coordinating Centre (NETSCC) Project Oversight Groups Guidance. Details of membership of the TSC and its Terms of Reference will be held on the Trial Master File (TMF).

#### Trial Management Group (TMG)

This will comprise all co-applicants, members of the NCTU, and Site Leads at each site. The TMG will be responsible for monitoring the progress of the study, addressing key issues that may arise and reporting to the Funder. Meetings will take place every 3 months, or more frequently if required. Those that coincide with the TSC meetings will meet a month before the TSC does.

#### Study team

A core team consisting of the CI and the TM will form the Study Team to monitor day-to-day progress. Wider study team members, including Site Leads (via video or phone-conference) and data management, will attend meetings where relevant to the phase of the study. This team will meet regularly to ensure all practical aspects of the trial are progressing well and identify potential issues as early as possible. Email discussion will also take place when appropriate.

#### Stakeholder forum

A Stakeholder Forum with representation from foster carers and professionals involved with children’s social care services will be convened. They will meet three times during the study, providing a way for the study team to communicate with the wider community, to follow policy development, to receive input into the design and delivery of the trials, and to support the dissemination programme. This Forum will have input from Patient and Public Involvement (PPI).

The TMG and the TSC will receive reports from the Stakeholder Forum.

### Composition of the data monitoring committee, its role, and reporting structure {21a}

It was agreed with the Funder that the study did not need a separate Data Monitoring Committee (DMC) because the intervention is not a psychological therapy, but rather a psychologically based training programme for professional foster carers. The Funder accepted the view that this required a different approach to a study evaluating a therapeutic intervention with a patient population presenting with a clinical need. Responsibilities of the DMC will therefore be managed by the TSC.

### Adverse event reporting and harms {22}

The Reflective Fostering Programme is a low-risk intervention. No specific risks or untoward incidents were reported during the development and pilot evaluation work. The intervention is not a medicinal product nor novel physiological or surgical procedure. The trial primary and secondary outcomes include safety outcomes (for example, placement stability). Therefore, no additional endpoints will be collected for safety (e.g. adverse events or serious adverse events) over and above the primary and secondary efficacy endpoints, other than an incident report from for those attending the Reflective Fostering Programme.

If carers in the intervention arm become distressed during the Reflective Fostering Programme sessions, one of the facilitators can attend to them using a break-out room (if online) or in a separate room (if groups run face-to-face) if it is not appropriate to remain in the general meeting, and the carer will be supported until resolution, stabilisation, or until it has been shown that the distress will not have any adverse impact and/or the study intervention is not the cause. At the end of the session, an incident report form will be completed and passed to the TM who will keep a log of such events. This intervention log will be shared with the TSC as part of safety monitoring.

If any risk disclosures occur during the Reflective Fostering Programme sessions, facilitators will discuss this with their practice supervisor or team manager, or escalate to the safeguarding lead at the relevant local authority. If any risk disclosures occur during focus groups or interviews in the Pilot phase and/or process evaluation the research team member will discuss these with the CI. If risk may be significant and/or imminent, the Site Lead(s) or local safeguarding officer would be contacted for further discussion and appropriate action. The Site Lead(s) will follow local procedures for dealing with safeguarding issues. The local authority holds ultimate safeguarding responsibility.

### Frequency and plans for auditing trial conduct {23}

NCTU staff will review data for errors and missing key data points. The trial database will also be programmed to generate reports on errors and error rates. The audit trail for the database will be monitored regularly for any unauthorised access. The TM will monitor the Investigator Site Files and outputs from data review. Essential trial issues, events, and outputs, including defined key data points, will be detailed in the trial Data Management Plan.

The frequency, type, and intensity of routine and triggered remote monitoring will be detailed in the Quality Management and Monitoring Plan (QMMP). The QMMP will also detail the procedures for review and sign-off of monitoring reports.

### Plans for communicating important protocol amendments to relevant parties (e.g. trial participants, ethical committees) {25}

The TM (or delegate) will be responsible for making amendments including updating the protocol and applying for ethical approval. The protocol will record the version history so that the most recent version can be identified and changes to the protocol will be recorded at the end of the document as they are made.

Substantial amendments that require review by the ethics committee will not be implemented until the committee grants a favourable opinion for the trial (amendments may also need to be reviewed and accepted by the governance team at the local authority before they can be implemented in practice at sites). All correspondence with the ethics committee will be retained in the TMF/Investigator Site File.

Once the amendments have received ethical approval, details of the amendments and any revised documentation will be circulated to the Funder, Sponsor, all sites, and trial participants where it impacts their involvement in the study.

### Dissemination plans {31a}

A Dissemination Policy will be written and submitted for approval to the TSC.

The TSC have responsibility for ensuring effective dissemination of the study results. On completion of the trial, the data will be analysed and tabulated and a Final Trial Report prepared for presentation to the Funder (NIHR).

Dissemination activity will take a range of formats: (1) publication in the NIHR PHR journal, social work journals, and/or other suitable peer-review journals; (2) results shared with participants via a study Newsletter, disseminated at regional events, and included in newsletters of relevant organisations; (3) the study team will host reports and blogs on the Anna Freud Centre’s Learning Network on latest evidence and research, and findings will also be shared via university repositories and social media; (4) foster carers and care-leavers will work with a creative arts team to disseminate findings to the wider public, possibly through a short film; and (5) each social care team involved will gain skills which can be used beyond the trial to support fostering skills.

If the trial establishes that the Reflective Fostering Programme is effective in improving health-related quality of life of children in care and is cost-effective, there is potential for the Programme to be rolled out nationally. Beyond that, there would be the potential to develop adaptations of the Programme, e.g. for carers of adolescents in foster care, for adoptive parents, or for those working in residential care.

## Discussion

There is increasing interest in the development of parenting programmes that promote reflective capacity, with a focus on improving the carer-child relationship. Until now, most parenting programmes available for foster carers have centred around teaching behaviour management skills and do not focus primarily on developing reflective capacity in carers.

The Reflective Fostering Programme offers a new approach to improving the lives and wellbeing of children in care, by teaching foster carers to understand their own feelings and in turn, to respond more effectively to the children in their care. This study will provide evidence of the effectiveness and cost-effectiveness of the Reflective Fostering Programme in a multi-site randomised control trial.

Due to the impact of the COVID-19 pandemic, a decision was made to delay the start of recruitment to the study for 3 months. In line with UK government restrictions on face-to-face contact in place at the time, and to ensure the safety and wellbeing of our participants and the research team, we developed a model of online delivery of both facilitator training and the Reflective Fostering Programme. The majority of usual care provided by local authorities has also been moved online. The 3-month delay on the start of recruitment and delivery of the Programme was used by the clinical team at AFNCCF to adapt the Reflective Fostering Programme to an online format, and to pilot this online programme (outside the current study) to ensure good translation of the Programme to online delivery.

Changes to the protocol required as a result of COVID-19 contingency plans have been incorporated into this study protocol. Government guidance will continue to be monitored, with the option of moving interventions (both Reflective Fostering Programme and usual support) back to face-to-face if and when it is appropriate to do so.

## Trial status

The trial is currently recruiting. Screening and recruitment is due to run from February 2021 to September 2022. This reflects a 3-month pause on the study in line with current COVID 19 restrictions on face to face contact to adhere to social distancing guidelines.

## Abbreviations

CI: Chief Investigator
TM: Trial Manager
UH: University of Hertfordshire
UCL: University College London
UEA: University of East Anglia
AFNCCF: Anna Freud National Centre for Children and Families
KCC: Kent County Council
KCL: Kings College London
NCTU: Norwich Clinical Trials Unit
CRF: Case Report Form
CTU: Clinical Trials Unit
HRQoL: Health-related quality of life
LA: Local authority
NICE: The National Institute for Health and Care Excellence
NIHR: National Institute for Health Research
PIS: Participant Information Sheet
PPI: Patient and Public Involvement
QALY: Quality-adjusted life year
RCT: Randomised controlled trial
SPQ: Site Profile Questionnaire
TSC: Trial Steering Committee
UK: United Kingdom
